# Genome-wide copy number variation detection in a large cohort of diverse horse breeds by whole-genome sequencing

**DOI:** 10.3389/fvets.2023.1296213

**Published:** 2023-11-22

**Authors:** Xiangwei Tang, Bo Zhu, Ruimin Ren, Bin Chen, Sheng Li, Jingjing Gu

**Affiliations:** ^1^College of Animal Science and Technology, Hunan Agricultural University, Changsha, China; ^2^Novogene Bioinformatics Institute, Beijing, China; ^3^Maxun Biotechnology Institute, Changsha, China

**Keywords:** copy number variations, CNV, CNVR, Vst, horse

## Abstract

Understanding how genetic variants alter phenotypes is an essential aspect of genetic research. Copy number variations (CNVs), a type of prevalent genetic variation in the genome, have been the subject of extensive study for decades. Numerous CNVs have been identified and linked to specific phenotypes and diseases in horses. However, few studies utilizing whole-genome sequencing to detect CNVs in large horse populations have been conducted. Here, we performed whole-genome sequencing on a large cohort of 97 horses from 16 horse populations using Illumina Hiseq panels to detect common and breed-specific CNV regions (CNVRs) genome-wide. This is the largest number of breeds and individuals utilized in a whole genome sequencing-based horse CNV study, employing racing, sport, local, primitive, draft, and pony breeds from around the world. We identified 5,053 to 44,681 breed CNVRs in each of the 16 horse breeds, with median lengths ranging from 1.9 kb to 8 kb. Furthermore, using *Vst* statistics we analyzed the population differentiation of autosomal CNVRs in three diverse horse populations (Thoroughbred, Yakutian, and Przewalski’s horse). Functional annotations were performed on CNVR-overlapping genes and revealed that population-differentiated candidate genes (*CTSL, RAB11FIP3,* and *CTIF*) may be involved in selection and adaptation. Our pilot study has provided the horse genetic research community with a large and valuable CNVR dataset and has identified many potential horse breeding targets that require further validation and in-depth investigation.

## 1 Introduction

Understanding how genetic variations affect phenotypes is an essential topic in genetic research. Copy number variations (CNVs), a form of common genetic variation in the genome, have attracted a great deal of research attention for decades. It is generally accepted to classify CNVs as duplication or deletion, depending on whether the number of copies increases or decreases, and to define CNVs as having a length greater than 1 kilobase (kb) to several megabase (Mb) (1, 2). In contrast to single nucleotide polymorphisms (SNPs), which are changes of a single base pair, CNVs often result in larger genetic effects than SNPs, such as alterations in gene dosage, gene structure, regulatory region, and expression. CNVs may therefore be significant contributors to variations in phenotypes and adaptations through evolution in horses during domestication and artificial selection (3–5).

In farm animals, a large amount of CNVs have been identified and linked to certain phenotypes (6). For decades, array comparative genomic hybridization (aCGH) microarrays and SNP chips have been the most prevalent techniques for detecting CNVs. Using whole-genome sequencing, unique and rare CNVs in populations can be captured more precisely and comprehensively due to technological advances in sequencing (7). In recent years, horse CNV research has also progressed. The graying of a horse’s coat, for instance, is caused by a CNV duplication on the intron of the *STX17* gene, which makes gray horses susceptible to melanoma (8). The CNVs in Y chromosome-linked genes were evaluated for their association with aberrant sexual development and sterility in stallions (9), and a 200 kb CNV homozygous deletion on horse chromosome 29 was thought to be associated with equine sex development disorders (10). CNVs in the MHC region on chromosome 20 were suggested to be associated with insect bite hypersensitivity in the Friesian horse population (11). Several CNV-overlapping candidate genes were discovered to be associated with thermal adaptation in Jinjiang horses (12). Recent CNV survey studies utilized European horse breeds (13), Criollo Argentino horses (14), South Korean horse breeds (15), and purebred Spanish horses (16). However, all of these studies mentioned above were conducted either using PCRs, microarrays, or SNP chips and to our best knowledge, only a few studies were conducted using whole-genome sequencing to detect CNVs in horse populations (17, 18).

In this study, we performed whole-genome sequencing on a large cohort of 97 horses from 16 horse populations to detect common and breed-specific CNV regions (CNVRs) across the entire genome. This is the largest number of breeds and individuals utilized in a whole genome sequencing-based horse CNV study, employing horse breeds with distinct breed characteristics such as racing, sport, local, primitive, draft, and pony breeds from all over the world. Using the *Vst* statistics, we also analyzed the population differentiation in autosomal CNVRs in our three key horse populations (Thoroughbred is an intensively artificially selected horse breed, especially for racing performance; Yakutian is a local horse breed free to roam the Siberian Far East; and Przewalski’s horse is a primitive horse represented the sister lineage of modern domestic horses) for every breed pair. CNVR-overlapping genes were functionally annotated and putative genes for selection and adaptation were suggested. Our pilot study has provided the horse genetic research community with a large and valuable CNVR dataset and has identified many potential horse breeding targets that require further validation and in-depth investigation.

## 2 Materials and methods

### 2.1 Whole-genome sequencing data overview and quality control

In this study, we collected 35 horse fresh blood samples and extracted DNA samples using the standard phenol-chloroform method. we performed high-coverage (average 25.55 × depth) whole-genome sequencing on 35 horses using Illumina HiSeq 4,000 sequencer (Illumina, Inc., San Diego, CA, United States) for 150 bp paired-end reads with the 350 bp short-insert libraries according to the manufacturer’s protocol. We also retrieved dozens of horse whole-genome sequencing datasets from public databases. In total, our dataset consisted of 97 horses of 16 horse breeds from around the world, each with its distinctive characteristics, including endurance breeds: Arabian (AB) and Akhal-Teke (AT), light draft horses: Franches-Montagnes (MON) and Friesian (FS), local breeds: Mongolian (MG), Jeju (JEJU), and Yakutian (YAK), pony breeds: Debao (DB) and Shetland pony (ST), primitive wild horse: Przewalski’s horse (PRZ), racing breeds: American Quarter horse (QT), Standardbred (STD), and Thoroughbred (TB), and sports breeds: Andalusian (AL), Criollo (CR) and Hanoverian (HAN). As described in our previous studies (19, 20), in brief, raw sequencing reads first underwent quality control using FastQC software,^1^ and then clean reads were aligned to the horse reference genome (EquCab3.0) using BWA (21).

### 2.2 Detection of CNVs and CNVRs

For each horse individual, CNVs were defined using CNVnator (22) with the bin size set to 100 bp based on the sequencing depth and filtered out the lengths less than 1 kb. Only CNVs obtained in two or more individuals in each population were retained, and CNVs that were located on unplaced scaffolds were removed. The overlapping CNVs with a 50% reciprocal overlap rate were merged into population CNVRs in 16 horse populations. The types of CNVRs were classified as duplication (Dup), deletion (Del) and mixed (within the same CNVR, some breeds have Dups while others have Dels) based on their copy numbers.

### 2.3 Comparison with published CNVRs

To validate the reliability and novelty of our study, the positions of detected CNVRs were compared with 11 published horse CNV studies (12, 13, 15, 16, 23–29) based on the EquCab3.0 reference genome.

### 2.4 Genomic selection signals based on CNVRs

The *Vst* statistic was used to access the population differential extent based on CNVRs between horse breeds. The *Vst* was calculated using the following equation: $Vst=\left(Vt-Vs\right)/Vt$ (30). The *Vt* denotes the total variance of copy number variations between the two populations. The *Vs* represents the mean variance of copy number variations within the population which is weighted by the corresponding population size. The pairwise *Vst* of CNVRs located on autosomes was calculated between TB, YAK, and PRZ. The top 1% of CNVR-based genomic selective signals were considered putative population-differentiated CNVRs.

### 2.5 Functional annotation and enrichment analysis of CNVRs

The CNVRs were annotated using ANNOVAR (31) according to their physical locations on the horse genome and classified as intergenic, intronic, exonic, downstream, upstream, splicing site, 3’UTR, and 5’UTR. The CNVRs were further annotated by searching the horse QTL database (The Animal QTLdb (32), https://www.animalgenome.org/cgi-bin/QTLdb/EC/index, accessed on 20 July 2023).

Genes whose gene bodies overlapped with candidate CNVRs were referred to as CNVR-overlapping genes. The Gene Ontology (GO) and Kyoto Encyclopedia of Genes and Genomes (KEGG) functional enrichment analysis of CNVR-overlapping genes were performed using DAVID (33) (https://david.ncifcrf.gov/tools.jsp last accessed on 12 August 2023).

## 3 Results

### 3.1 Detection of horse CNVRs

In total, 6195.88 Gb clean data from whole-genome sequencing of 97 horses were analyzed. The average sequencing depth was 25.55× and the coverage rate of at least 4× was 93.28% ensuring the reliability of CNV detection (Supplementary Table S1). CNVs were first called for each horse (Supplementary Table S2), and the overlapping CNVs from the same population were then merged into breed CNVRs. We identified 5,053 to 44,681 breed CNVRs in each of the 16 horse breeds with median lengths from 1.9 kb to 8 kb (Table 1). The TB had the highest number of CNVRs accounting for 13.78% of the total genome length, while JEJU had the least number of CNVRs accounting for 6.21% of the total genome length. We found 2,036 common CNVR-harboring genes shared by 16 horse breeds. Those common CNVR-harboring genes were mainly involved in immune response (GO:0019882, ecb05150, GO:0002504, GO:0002503, GO:0019886, ecb05340), metabolism (ecb04940, ecb01100) and neurotransmission (GO:0007268, GO:0050804, ecb04080, ecb04724, GO:0098887) (Supplementary Table S3). We further searched for CNVR-harboring genes specific to each breed in the expectation of understanding whether CNVs might shape breed characteristics. We obtained gene function enrichment results from 6 horse breeds, and no results were obtained from the remaining 10 breeds due to too few breed-specific CNVR-harboring genes (Supplementary Table S4 and Supplementary Figure S1). The TB-specific CNVR-harboring genes were involved in lipid metabolism and proteoglycan biosynthesis. The YAK-specific CNVR-harboring genes were involved in complement and coagulation cascades and one-carbon metabolic processes. The QT-specific CNVR-harboring genes were mainly involved in primary immunodeficiency and neuroactive ligand-receptor interaction. The PRZ-specific CNVR-harboring genes were involved in immune response and cytokine–cytokine receptor interactions. The MG-specific CNVR-harboring genes were involved in the detection of chemical stimuli involved in the sensory perception of bitter taste and the inflammatory response. The JEJU-specific CNVR-harboring genes were involved in the positive regulation of osteoblast differentiation.

**Table 1:** The general statistic of copy number variation regions (CNVRs) of 16 horse breeds.

Breed Abbr.	Sample (*n*)	CNVR (*n*)	Duplication (*n*)	Deletion (*n*)	Mixed (*n*)	Median length (bp)	Total Length (bp)	CNVR Coverage (%)^a^
AB	5	7,208	4,540	7,804	1,041	3,000	91,830,026	3.81%
AL	4	6,932	4,412	5,546	695	3,700	91,910,526	3.82%
AT	5	7,435	4,577	8,009	1,133	2,800	91,746,812	3.81%
CR	2	6,369	2,996	4,357	362	4,500	88,872,468	3.69%
DB	5	8,939	5,888	8,200	1,508	3,300	92,993,095	3.86%
FS	5	7,601	4,579	8,430	987	2,900	85,405,012	3.55%
HAN	4	6,302	3,708	6,329	851	3,500	95,100,747	3.95%
JEJU	2	5,053	2,369	2,545	338	8,000	149,649,478	6.21%
MG	5	10,330	5,875	8,523	2,393	3,700	115,915,549	4.81%
MON	5	17,136	4,438	33,666	1,381	1,900	122,939,957	5.10%
PRZ	10	6,770	3,657	7,478	1,330	3,700	83,850,677	3.48%
QT	7	22,079	8,480	24,902	8,873	2,402	151,000,707	6.27%
ST	3	17,730	8,033	20,025	5,140	2,400	136,584,714	5.67%
STD	4	12,953	4,723	12,301	2,598	2,601	121,737,989	5.05%
TB	22	44,681	37,809	57,957	12,740	3,200	331,917,190	13.78%
YAK	9	9,407	7,355	10,371	2,301	3,301	115,508,128	4.79%

a CNVR coverage in the horse genome was calculated using the total length of CNVRs divided by the horse genome length for each horse breed.

**Figure 1:**
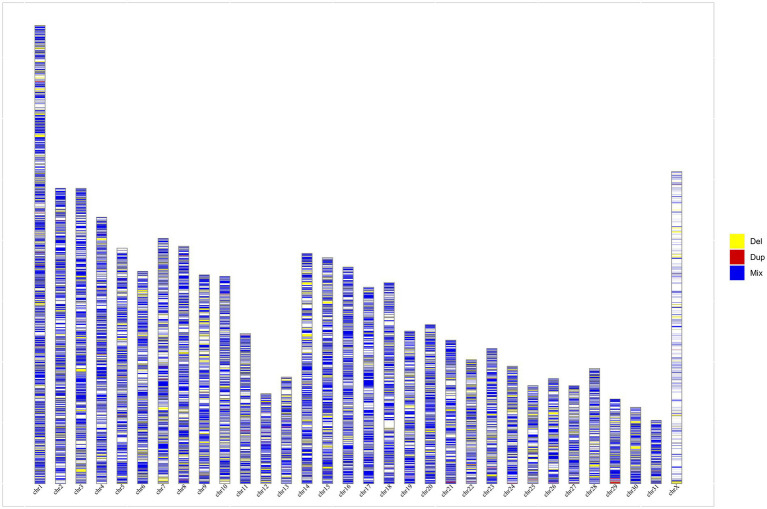
The distribution of CNVRs by length throughout the horse genome. Yellow, red, and blue represent deletion (Del), duplication (Dup), and Mixed (Del and Dup), respectively.

By merging the overlapping CNVs in all 16 horse breeds, we obtained 43,838 population CNVRs including 517 duplications, 12,275 deletions, and 31,046 mixed events (Supplementary Table S5). The distribution of CNVRs by length was uneven throughout the horse genome (Figure 1) with a median length of 9.3 kb. The largest number of CNVRs with lengths of 10 kb–20 kb accounted for 18.13%. The second and third most abundant CNVRs were 2 kb–4 kb and 4 kb–6 kb in length, accounting for 14.21 and 12.18%, respectively. The number of CNVRs longer than 0.4 Mb is rare accounting for only 1.24%. Population CNVRs were further annotated according to their position on the horse genome as intergenic, exonic, intronic, upstream, downstream, 3’UTR, and 5’UTR. There were 35.8% of CNVRs located intergenic, 35.9% of CNVRs located exonic, and 15.9% of CNVRs located intronic (Supplementary Figure S2).

### 3.2 Comparison with published reports and horse quantitative trait loci database

We compared the population CNVRs obtained from 97 horses, with the results of 11 published studies of horse CNVs, and found 1,973 CNVRs overlapped with the results of the published studies (Supplementary Table S6). To further reveal the relatedness of CNVRs to horse traits, the obtained CNVRs were searched in the horse QTL database. We found that 5,692 CNVRs overlapped with 58 horse QTL traits (Supplementary Table S7). The top 5 categories of horse QTLs that overlapped with CNVRs were osteochondrosis and osteochondrosis dissecans (1,311 CNVRs), recurrent airway obstruction (989 CNVRs), navicular bone morphology (617 CNVRs), recurrent exertional rhabdomyolysis (378 CNVRs), and equine sarcoids (300 CNVRs).

### 3.3 CNVR-based population differentiation

**Figure 2:**
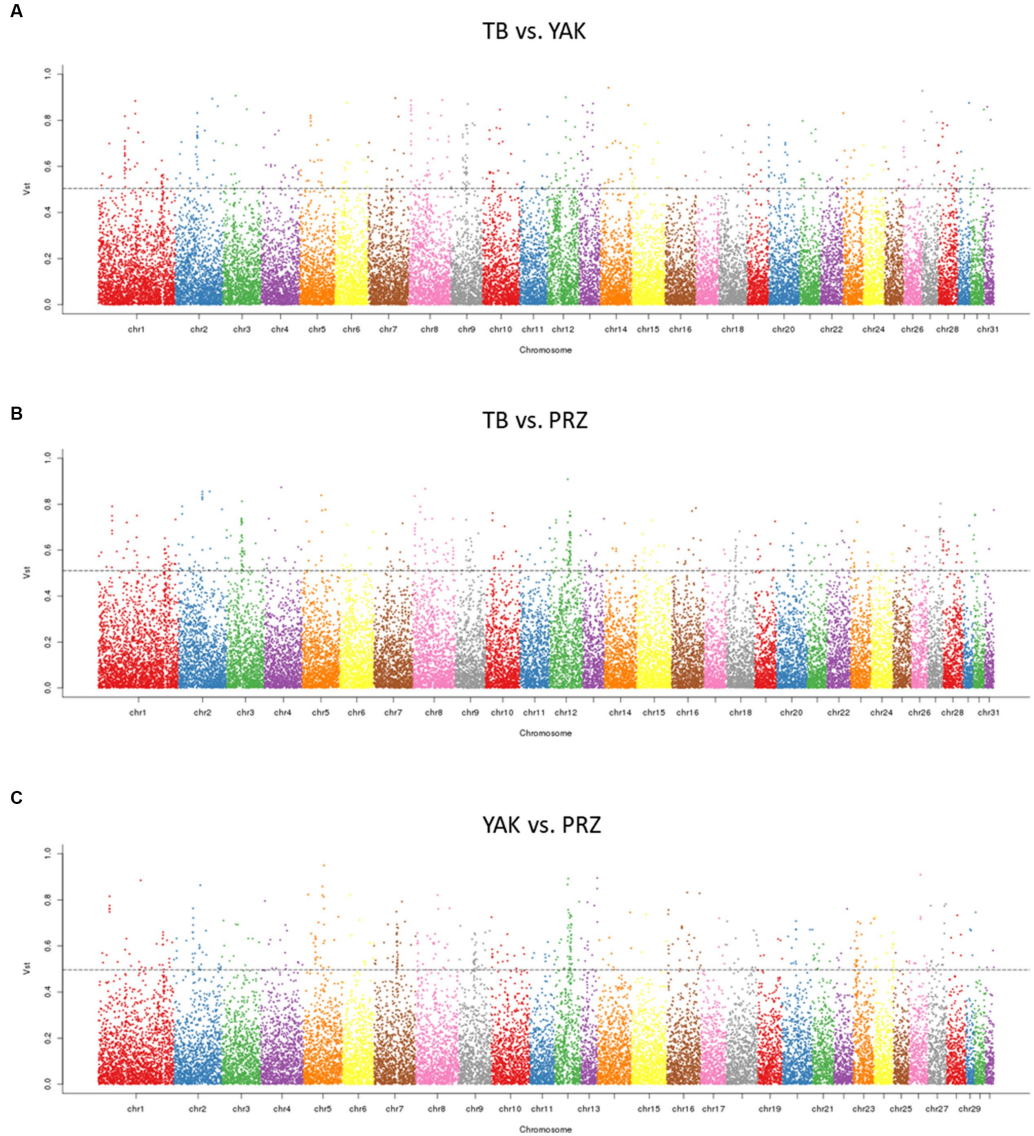
Manhattan plots of genome-wide pairwise *Vst* for CNVRs in three comparisons **(A)** TB vs. YAK **(B)** TB vs. PRZ **(C)** YAK vs. PRZ among autosomal chromosomes. The dotted line represents the top 1% *Vst* threshold.

Using *Vst* statistics, we examined the CNVR-based population differentiation among three key horse populations. The pairwise *Vst* was calculated between TB and YAK, TB and PRZ, and YAK and PRZ. The top 1% value of *Vst* was determined as a threshold value for the three comparisons based on the empirical distribution and the top 1% values of *Vst* were about 0.504 (TB vs. YAK), 0.511 (TB vs. PRZ), and 0.496 (YAK vs. PRZ). The number of CNVRs within the 1% highest *Vst* values was 243 in the TB vs. YAK pair, 280 in the TB vs. PRZ pair, and 247 in the YAK vs. PRZ pair. As shown in Figure 2 and Supplementary Table S8, the different CNVRs among the populations were unevenly distributed on the horse chromosome.

Between TB and YAK, 401 CNVR-overlapping genes showed significant differentiation and were mainly involved in olfactory transduction (ecb04740), fc gamma R-mediated phagocytosis (ecb04666), osteoclast differentiation (ecb04380), positive regulation of mitochondrial fusion (GO:0010636), negative regulation of gene expression (GO:0010629) and protein phosphorylation (GO:0001933). Between TB and PRZ, 370 CNVR-overlapping genes showed significant divergence and were mainly involved in defense/immunity response (GO:0006952, GO:0051673, GO:0019731, GO:0071222, and GO:0002227), hyaluronan metabolic process (GO:0030212) and intracellular protein transport (GO:0006886). Between YAK and PRZ, 338 CNVR-overlapping genes showed significant population differentiation and were mainly involved in signal transduction (GO:0007165) and the hippo signaling pathway (ecb04390) (Supplementary Table S9).

It was worth noting that only three CNVR-overlapping annotated genes were presented in both the TB vs. YAK and TB vs. PRZ comparisons, and these genes may be associated with TB’s athletic ability (Supplementary Figure S3). The types of CNVR within three genes were deletions in the exonic region. Peroxiredoxin-like 2A (*PRXL2A*) regulates osteoclast differentiation (34, 35). The CNVR within *PRXL2A* was a deletion type in TB, YAK, and PRZ with a length of 47,699 bp and was located in the exonic region on ECA1 from 89,585,201 to 89,632,900 bp. Phosphoinositide-3-kinase regulatory subunit 2 (*PIK3R2*) is involved in cellular glucose homeostasis (36). The CNVR within *PIK3R2* was not detected in PRZ, whereas a deletion type was detected in TB and YAK with a length of 235,299 bp and was located in the exonic region on ECA21 from 3,337,101 to 3,572,400 bp. Death-associated protein kinase 1 (*DAPK1*) is associated with myocardial injury (37). The CNVR within *DAPK1* was a deletion type with a length of 24,999 bp and was located in the exonic region on ECA23 from 2,866,801 to 2,891,800 bp. Those deletions of gene exons may result in truncated proteins, with loss of protein functions.

## 4 Discussion

Horses domesticated around 5,500 years ago, have played a crucial role in human society (38). Due to variations in social structure and geography, over 600 distinct horse breeds have been developed to meet the diverse needs of humans (39). The release of the horse reference genome (40, 41) has accelerated investigations into the genetic mechanisms underlying complex traits in horses. To date, many studies have focusing on the discovery of CNVs in horses, but the vast majority of these studies have employed SNP chips or microarrays, resulting in a lower resolution of detected CNVs (42).

In the present study, we used the high-depth whole-genome sequencing data of 97 horse individuals from 16 global horse breeds to detect CNVs with a minimum resolution of 100 bp. We identified 43,838 population CNVRs, of which 70.8% were classified as mixed type, demonstrating the high prevalence and polymorphic nature of CNV in horse populations. About 35.8% of CNVRs were located in intergenic regions, demonstrating the regulatory potential of CNVs. By comparing our results to more than a dozen published studies on horse CNV, we found that about 95% of the CNVRs were found for the first time in our study. This indicated that resequencing the entire genome at high depth using a large number of individuals and breeds can detect breed-specific CNVRs more frequently.

Olfactory receptors (ORs) belong to G protein-coupled receptors (GPCRs), which are predominantly expressed in nasal epithelial cells (43, 44). In addition to recognizing various odors to assist in feeding (45), ORs are widely distributed in other non-olfactory tissues (e.g., testis, liver, heart, and kidney) (46–49) and germ cells (sperm and oocytes) (50, 51) and play important roles in glucose and lipid metabolism (52), reproduction (51), and pathogen recognition (53). In our results, 330 OR genes harbored with population CNVRs accounted for 30% of all OR genes on the horse genome (54) suggesting potential gene targets for selection.

By comparing population CNVRs to the horse QTL database, 58 equine QTLs were discovered to overlap with CNVRs. The top categories of horse QTLs that overlapped with CNVRs were osteochondrosis and osteochondrosis dissecans. Several CNVRs harboring genes, such as B cell receptor-associated protein 29 (*BCAP29*) and Wnt family member 9A (*WNT9A*), are located in osteochondrosis QTL regions. *BCAP29* is associated with osteoblast differentiation (55, 56). The CNVR within *BCAP29* was a deletion type with a length of 16,399 bp and was located in the exonic region on horse chromosome 4 (ECA4) from 7,561,901 to 7,578,300 bp. *WNT9A* plays a key role in embryonic skeletal joint development (57, 58) and mice deficient in Wnt9a are susceptible to sporadic osteoarthritis as they age (59). The CNVR within *WNT9A* was also a deletion type with a length of 59,199 bp and was located in the exonic region on ECA14 from 94,378,801 to 94,438,000 bp. These identified CNVR-overlapping genes provided candidate markers for future horse breeding.

Selective sweep analysis can reveal potential genomic regions that have been subjected to artificial and natural selection during domestication and acclimation. By conducting pairwise *Vst* statistics, we further screened for significantly different CNVRs among TB, YAK, and PRZ horse populations. The three horse breeds were selected based on their distinguishing characteristics (TB: racing horse; YAK: local horse; PRZ: primitive horse) and sufficient numbers of individuals of each breed in our data set. In TB, YAK, and PRZ, only three CNVR-overlapping protein-coding genes (cathepsin L, *CTSL*; RAB11 family interacting protein 3, *RAB11FIP3*; and cap binding complex dependent translation initiation factor, *CTIF*) showed significant pairwise differences in all three comparisons simultaneously. *CTSL* encodes a lysosomal cysteine proteinase with a crucial function in intracellular protein catabolism and is involved in antigen processing (60), bone remodeling (61), and cardiac morphology (62). *RAB11FIP3* is a member of the Rab GTPase family and regulates intracellular transport vesicle formation, targeting, and fusion (63). It is also involved in regulating T-cell activation (64). *CTIF* is a component of the translation initiation complex involved in protein translation (65) and an SNP within the *CTIF* gene is associated with hearing loss (66). These CNVR-overlapping genes with inter-population copy number differentials suggested that CNVs may shape the genetic background of different horse breeds.

## 5 Conclusion

In this study, we made a comprehensive analysis using high-depth whole-genome sequencing technology of 97 horses belonging to 16 global horse breeds. We defined common and breed-specific CNVRs and further analyzed the possible functions of CNVR-overlapping genes using enrichment analysis and QTL database searches. Based on pairwise *Vst* statistics, we examined the CNVR-based population differentiation among three key horse populations and revealed potential genomic regions that might be under selection. Our pilot study provided a large and valuable CNVR data set for the horse genetic research community and suggested many candidate targets for horse breeding.

## Data availability statement

The datasets presented in this study can be found in online repositories. The names of the repository/repositories and accession number(s) can be found in the article/Supplementary material.

## Ethics statement

The animal study was approved by Biomedical Research Ethics Committee of Hunan Agricultural University. The study was conducted in accordance with the local legislation and institutional requirements.

## Author contributions

XT: Formal analysis, Writing – original draft, Writing – review & editing. BZ: Formal analysis, Writing – original draft, Writing – review & editing. RR: Resources, Software, Writing – review & editing. BC: Resources, Software, Writing – review & editing. SL: Conceptualization, Data curation, Formal analysis, Investigation, Methodology, Resources, Software, Supervision, Validation, Visualization, Writing – original draft, Writing – review & editing. JG: Conceptualization, Data curation, Formal analysis, Funding acquisition, Investigation, Methodology, Project administration, Resources, Software, Supervision, Validation, Visualization, Writing – original draft, Writing – review & editing.

## Footnotes

^1^ http://www.bioinformatics.babraham.ac.uk/projects/fastqc/
