# Supplementary material for: Genome-wide copy number variation detection in a large cohort of diverse horse breeds by whole-genome sequencing
Source: Front Vet Sci. 2023 Nov 22;10:1296213. doi: 10.3389/fvets.2023.1296213 (PMC10710158; doi:10.3389/fvets.2023.1296213)
Supplement: Supplementary file 1 [file Data_Sheet_1.ZIP › Supplementary Figures and Tables_revised/Supplementary Figure 1-3.docx]

**Supplementary Figures:**

**Supplementary Figure 1** The number of the common and breed-specific CNVR-harboring genes. The number of common CNVR-harboring genes shared by 16 horse breeds is 2036. The numbers at the top of each bar indicated the number of breed-specific CNVR-harboring genes. Horse breed abbreviation: Arabian (AB), Andalusian (AL), Akhal Teke (AT), Criollo (CR), Debao (DB), Friesian (FS), Hanoverian (HAN), Jeju (JEJU), Mongolian (MG), Franches-Montagnes (MON), Przewalskii (PRZ), Quarter horse (QT), Shetland pony (ST), Standardbred (STD), Thoroughbred (TB), Yakutian (YAK).

**Supplementary Figure 2** Pie chart of the annotated population CNVRs. Population CNVRs were further annotated according to their position on the horse genome as intergenic, exonic, intronic, upstream, downstream, 3’UTR, and 5’UTR. There were 35.8% of CNVRs located intergenic, 35.9% of CNVRs located exonic, and 15.9% of CNVRs located intronic.


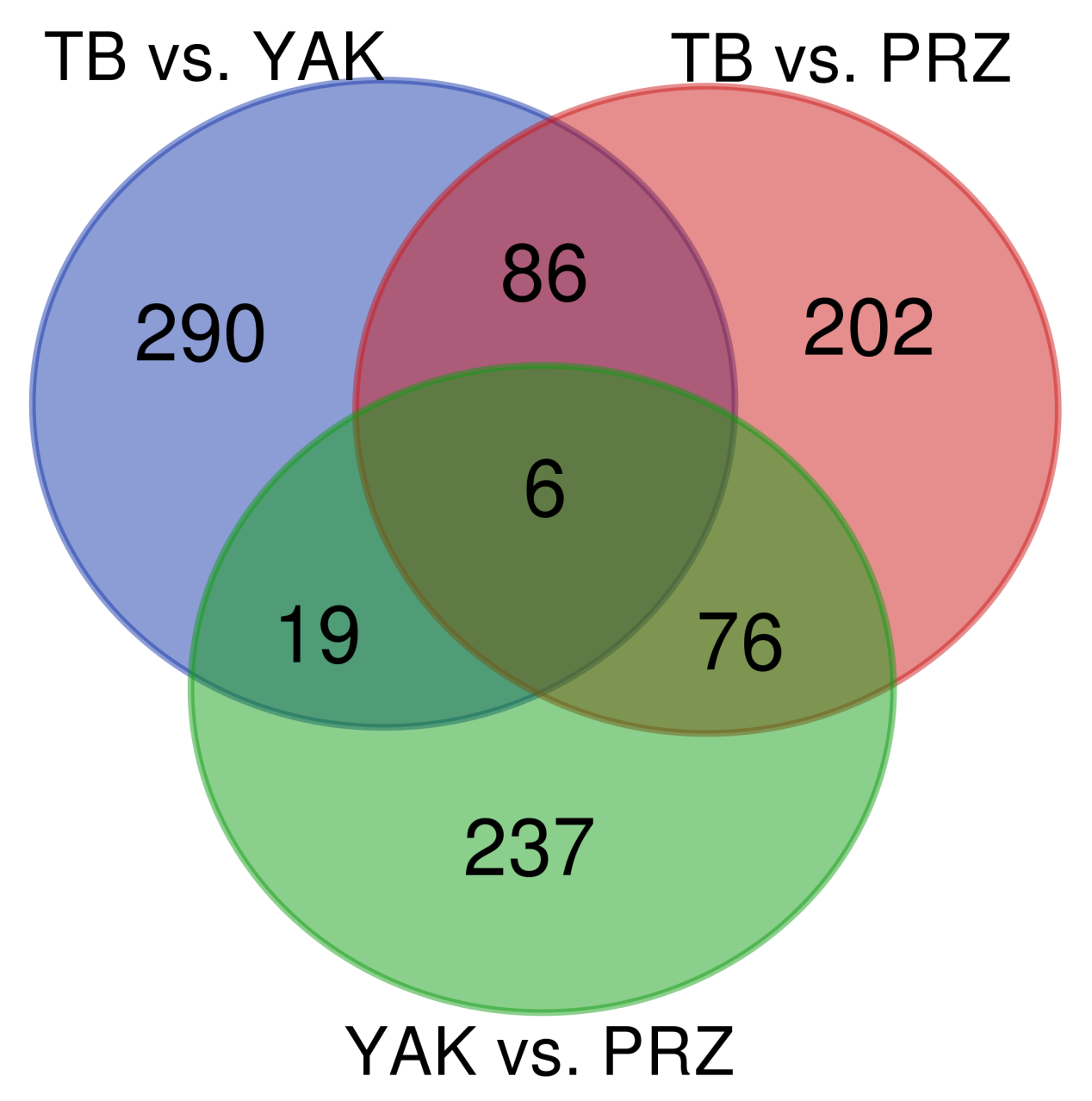


**Supplementary Figure 3** The Venn diagram represents the shared number of CNVR-harboring Ensembl genes between pairwise comparisons of TB vs. YAK, TB vs. PRZ, and YAK vs. PRZ.
